# A new way to protect privacy in large-scale genome-wide association studies

**DOI:** 10.1093/bioinformatics/btt066

**Published:** 2013-02-14

**Authors:** Liina Kamm, Dan Bogdanov, Sven Laur, Jaak Vilo

**Affiliations:** ^1^Institute of Computer Science, University of Tartu, Liivi 2, Tartu 50409, Estonia, ^2^Software Technology and Applications Competence Center, Ülikooli 2, Tartu 51003, Estonia and ^3^Cybernetica AS, Ülikooli 2, Tartu 51003, Estonia

## Abstract

**Motivation:** Increased availability of various genotyping techniques has initiated a race for finding genetic markers that can be used in diagnostics and personalized medicine. Although many genetic risk factors are known, key causes of common diseases with complex heritage patterns are still unknown. Identification of such complex traits requires a targeted study over a large collection of data. Ideally, such studies bring together data from many biobanks. However, data aggregation on such a large scale raises many privacy issues.

**Results:** We show how to conduct such studies without violating privacy of individual donors and without leaking the data to third parties. The presented solution has provable security guarantees.

**Contact:** jaak.vilo@ut.ee

**Supplementary information:** Supplementary data are available at *Bioinformatics* online.

## 1 INTRODUCTION

Genome-wide association studies (GWAS) are one of the driving reasons behind the formation of nationwide and privately funded gene banks. Many chronic diseases and various cancer types are known to have genetic disposition factors (Chakravarti and Little, 2003; Lander and Schork, 1994). Although many underlying genetic signatures have been successfully identified for Mendelian disorders (Hamosh *et al.*, 2005), not many genetic risk factors for complex diseases have been discovered and confirmed. GWAS have identified some risk factors for type II diabetes (Prokopenko *et al.*, 2008) and for a few other common diseases (Manolio *et al.*, 2008; Wellcome Trust Case Control Consortium, 2007). GWAS have been modestly successful in pharmacogenetics (Grant and Hakonarson, 2007) and cancer research (Varghese and Easton, 2010). The size and structure of a study cohort are the main limiting factors in such studies, as the individual impact of genomic differences is usually small. Larger sample sizes increase the sensitivity of statistical tests and make it possible to apply a wide range of data-mining methods (Moore *et al.*, 2010; Szymczak *et al.*, 2009).

Ideally, studies should use nationwide and continent-wide patient cohorts. Formation of such cohorts is becoming feasible as genotyping costs are rapidly decreasing (Hayden, 2010; Pettersson *et al.*, 2009). In addition to nationwide biobanks, e.g. the UK Biobank, several personal genomics companies, such as 23andMe and Navigenics, already possess large and diverse patient cohorts. Biobanks are also forming large collaboration networks, such as P3G and HuGENet, to combine their patient cohorts and improve study quality. Privacy of individual gene donors is one of the biggest concerns in such projects. In many countries, genotype data are classified as sensitive data that can be handled by complying with specific restrictions, e.g. HIPAA in the USA and the Data Protection Directive in the European Union. These restrictions are justified, as a leak of genetic information can cause genome-based discrimination when more health-related patterns have been discovered.

Standard anonymization methods are not applicable to genotype data, as the data themselves are an ultimate identity code. Only 30–80 out of 30 million single-nucleotide polymorphisms (SNPs) are needed to uniquely identify a person (Lin *et al.*, 2004). Moreover, the size of online genotype databases for genealogy studies, such as SGMF and YHRD, has made re-identification of anonymized genotype data a real threat (Gymrek *et al.*, 2013). Re-identification attacks (Malin and Sweeney, 2000, 2002) based on combining inferred phenotypes with public data become practical, as the list of known associations between genotype and phenotypic traits (Hindorff *et al.*, 2009) evolves. Finally, Homer *et al.* (2008) showed that even aggregated pools of genomic data can leak private information. Although follow-up studies (Visscher and Hill, 2009) softened initial claims, the threat remains.

These findings created a debate whether one can promise privacy of genotype data in consent forms at all (P3G Consortium *et al.*, 2009). In the following, we show how to set up an infrastructure where the genotype data can be stored and processed so that none of the peers involved in the process can reconstruct the data, and thus the risk of accidental leaks and malicious data abuse is greatly reduced. The data analysis algorithms are executed in an oblivious manner so that only the desired outcome is revealed to the user and nothing else. Differently from well-known data perturbation and masking techniques (Machanavajjhala *et al.*, 2007; Sweeney, 2002), security guarantees are cryptographic. These guarantees depend on the computational complexity of well-established mathematical problems and not on the background knowledge of potential attackers. As such, the presented methodology is applicable to protecting biobanks and other medical databases.

## 2 APPLICATION SCENARIOS

### 2.1 Privacy threats in medical studies

There are four principal groups of stakeholders in a typical medical study: data donors, data collectors, data analysts and supervisory organizations. Data donors consent to give their tissue samples and record other types of medical data, e.g. various questionnaires covering health issues. Data collectors are responsible for gathering and storing the data and keeping the donors’ confidentiality. The baseline requirements are forced by the laws, e.g. by the Genetic Information Nondiscrimination Act of 2008 in the USA. However, more stringent privacy guarantees can reduce data donors’ fears about data abuse and increase the participation rate.

Data collectors and supervisory organizations must guarantee that the data analysts (researchers) meet privacy restrictions. The confidentiality problem is somewhat smaller if the data analysts are from the same institution as the data collectors. If the analysts are not part of the organization, usually confidentiality agreements are signed between the parties. In most cases, collected data are stored in several databases so that direct personal information is not accessible to researchers. Instead, each patient gets a (pseudo) random barcode that links different databases together (De Moor *et al.*, 2003). On rare occasions, databases are merged either to identify specific persons or to form datasets needed for medical research.

All such solutions provide only partial security guarantees for data donors. Even if genetic data are stored separately and are not directly accessible, they must be (partially) released before the actual data analysis is carried out. Hence, a single fault by a data analyst or an insider attack might effectively obliterate all privacy guarantees. Therefore, compromises in confidentiality are needed for the creation of worldwide data banks for the scientific community.

These privacy issues are often alleviated with data perturbation and agglomeration techniques. For instance, the National Institutes of Health in the USA published the ratio of SNP alleles of various case–control studies (Couzin, 2008) because it is essentially impossible to split a DNA mixture back to individual genotypes. However, it turned out that it is possible to detect whether a specific person is in the mixture or not (Homer *et al.*, 2008). As case–control groups are often based on sensitive information, the data had to be removed.

Such unexpected security breaches are common for *ad hoc* perturbation or agglomeration methods because one often overlooks the effect of potential background knowledge to security. Although for certain problems perturbation and agglomeration methods can provide provable security, their overall applicability is limited and there are many known impossibility results (Dwork, 2011).

### 2.2 A novel solution based on distributed storage

**Fig. 1.**
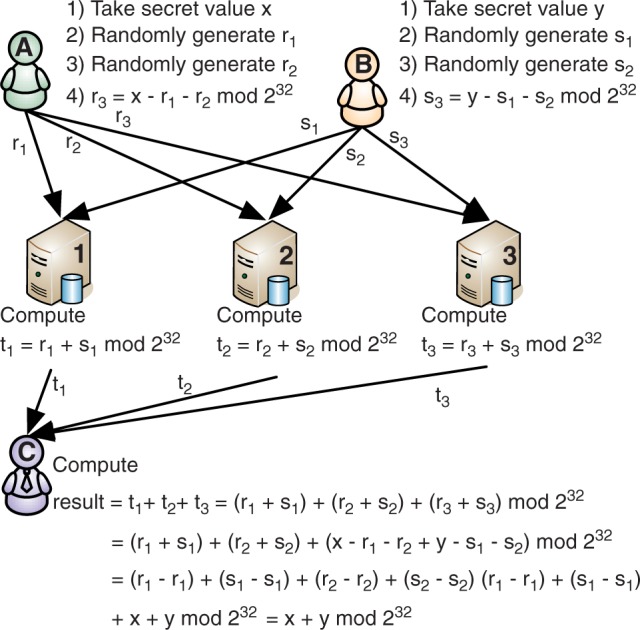
This figure illustrates how players A and B use a *3-out-of-3* additive secret sharing scheme to distribute two 32-bit integer values *x* and *y* to shares. The shares are sent to three servers that use the homomorphic property of the scheme to securely compute the sum of *x* and *y*. The shares of the sum are sent to player C, who reconstructs the result

In this work, we propose a data collection system where sensitive data are secret shared among several independent entities. In brief, secret sharing assures that each party gets completely random-looking data. However, when all parties pool the data together sensitive values can be restored (see Fig. 1 for an example of how secret sharing works). Depending on the exact nature of the used secret sharing scheme, the privacy of shared values is preserved even if some of the parties holding shares collude (discussed further in the methods section). Hence, data can be shared without the fear of unexpected disclosure. Moreover, with the use of specific multi-party computation techniques, computations on secret shared data can be carried out without leaking any information (Bogdanov *et al.*, 2008; Damgård *et al.*, 2009). As a result, one can deploy a distributed computation environment that securely collects the data, does oblivious computations and returns the desired end results. Such systems have been successfully used for securing auctions (Bogetoft *et al.*, 2009) and analyzing financial data (Bogdanov *et al.*, 2012).

There are other cryptographically secure computation techniques, e.g. (fully) homomorphic encryption. However, these techniques are significantly slower and less feasible on the large genome databases.

**Fig. 2.**
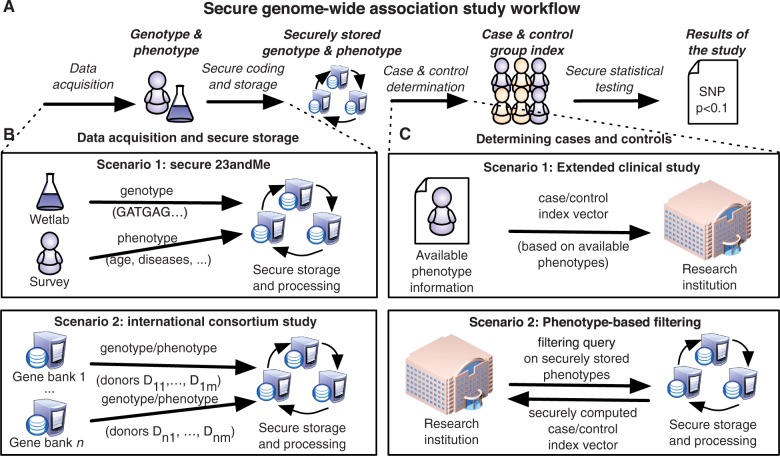
Secure GWAS consists of three major stages: data acquisition, formation of case–control groups and statistical testing. Panel (**A**) depicts how these three stages are linked. Data are gathered and sent in securely coded shares to be stored. For statistical tests, case and control info is securely coded and applied to the securely stored data so that statistical analyses can be carried out. Panel (**B**) describes two alternative scenarios that can be used for secure storage of genotype and phenotype data. Scenario 1 depicts a situation where genotype data are entered into secure storage by the wetlab and phenotype data are entered by the donors themselves. Scenario 2 depicts a case where different gene banks send selected genotype and phenotype data to secure storage so they can make joint analyses on more data. Panel (**C**) describes how case and control groups can be formed. In the simplest case, researches have unrestricted access to phenotype data and can thus form case and control groups by themselves. In more complex settings, researchers do not have rights to access phenotype data, and hosts must use secure multi-party computations to construct case and control groups based on inclusion criteria

Figure 2 depicts the overall workflow of secure GWAS. The core of such a system consists of three or more dedicated data centers (*hosts*) that are assumed to be independent organizations. For a worldwide study, these can be biobanks of different countries, regulatory authorities and patient interest groups. None of them have to be unconditionally trusted as long as too many of them do not collude with others. In particular, a successful attack against one or two of the data centers leaks no information and there even exists a recovery procedure from such attacks.

Genomic data are entered into the system by primary data collectors, e.g. wetlabs or different biobanks who collect and process the biological samples and who want to make joint analyses on shared data. For this operation, standard clinical protocols for genotyping are sufficient for security. At the end of the stage, the data are secret shared and transferred securely to the hosts.

Clinical data can be entered into the system either by the data donors themselves or by primary data collectors. The data are sent to the secure phenotype database in secret shared form to ensure confidentiality. In the analysis stage, the data analyst specifies the algorithm to be run on the secret shared data and waits for the results. For GWAS, the analyst first forms case and control groups. Next, the algorithm computes the necessary statistics and, finally, it releases the loci that show a statistically significant differentiation according to the specified case–control groups. If the analyst wants to further examine a secured input value, all parties hosting the system must agree to disclose the respective shares.

### 2.3 Potential advantages and drawbacks

We acknowledge that standard security measures are sufficient when the data are collected and analyzed by a single organization. Still, long-term projects can benefit from distributed data storage. First, a break-in into a data center yields no usable information. Second, splitting the data among independent organizations gives additional guarantees for the data donors, e.g. participants of commercial studies have no way of knowing what happens to their data if the project goes bankrupt. If one core center belongs to the state, participants know with greater certainty that their data cannot be abused. Third, other sources of private data can be incorporated into the analysis without privacy leaks. In particular, medical institutions can use their patient records to enhance analysis. The proposed solution provides a way to conduct the analysis so that neither the gene bank nor the medical institution releases their data.

The benefits of our approach are evident in collaborative studies between independent biobanks. As nothing beyond the desired test results are revealed during the computation, the solution provides superior privacy guarantees compared with alternatives based on meta-analysis techniques (Wolfson *et al.*, 2010), where each biobank first computes local summaries that are collaboratively merged into a final result. As a result, only a few summary values are disclosed. However, it is impossible to tell what exactly can be inferred about concealed values. Moreover, leakages of individual studies can cumulate as in DNA pools, where aggregation of minuscule effects on SNP frequencies allows us to make strong conclusions.

The biggest technical drawback of our solution is computational efficiency. As the data are secret shared between core centers and each oblivious computation step requires network communication, secure algorithms are several magnitudes slower than their insecure counterparts. Hence, we set up a controlled experiment to show that GWAS are feasible in our setting.

Finally, note that legal restrictions can form a major obstacle, as secret sharing is not as widely used as data encryption. However, legal issues are out of our scope as we analyze only the technological feasibility and potential advantages.

## 3 METHODS

### 3.1 Single-point analysis

The first step in GWAS is splitting individuals into case and control groups. These groups are formed based on phenotypic traits, such as presence and severity of disease. Although all four nucleotides can be located in a SNP site, it is common to consider two alleles, where the first one corresponds to the reference sequence and the other represents potential mutations. Table 1 depicts the 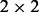 contingency table for allele counts in case and control groups, where an allele is counted twice if it is present in both DNA strands (is homozygous). The test statistic for the standard 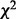 test is expressed as
(1)
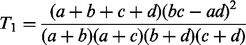

and the test statistic for equiproportionality of the allele A in both groups is
(2)
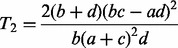

For reasonable sample sizes, both test statistics are distributed according to 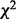 distribution with one degree of freedom. See the work of Visscher and Hellard (2003) for further details.

**Table 1.** Contingency table for the standard 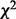 test

Group	Allele A	Allele B
Cases	*a*	*c*
Controls	*b*	*d*

These tests are accurate if the Hardy–Weinberg equilibrium condition is satisfied for a particular SNP, whereas the Cochran–Armitage test for trend can be used without this assumption. First, one must assemble the 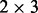 contingency table depicted in Table 2 and then compute the tests statistic as
(3)
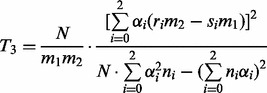

where the weights 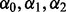 are chosen according to the suspected influence mechanism (usually 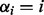 is an appropriate choice). As before, *T*_3_ is approximately distributed according to 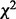 distribution with one degree of freedom (Armitage, 1955; Sasieni, 1997).

**Table 2.** Contingency table for the Cochran–Armitage test for trend

Group	Allele AA	Allele AB	Allele BB	Total
Cases	*r*_0_	*r*_1_	*r*_2_	*m*_1_
Controls	*s*_0_	*s*_1_	*s*_2_	*m*_2_
Total	*n*_0_	*n*_1_	*n*_2_	*N*

Transmission disequilibrium test (TDT) is applicable only if the data consist of parent–child trios. The test measures whether one homozygous genotype is more over-represented than the other among affected children with heterozygous parents. For that we must first select trios where both parents have heterozygous genotype. Let *u* be the count of AA and *v* be the count of BB genotypes among children. Then the corresponding statistic
(4)
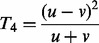

is again approximately distributed according to 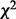 distribution with one degree of freedom (Spielman *et al.*, 1993). Compared with previous tests, TDT is less sensitive to sampling artefacts but it also requires more structured data.

### 3.2 Essentials of secure computing using secret sharing

A secure computation program is similar to a standard computer program. The difference between the two is in how the data are stored and processed. In a standard program, all values are processed publicly, whereas in a secure computation program, it is possible to specify which values are publicly visible and which are stored using techniques like secret sharing. These values can be used in computations without leaking their contents.

In our proposed solution, the data are secret shared between three or more hosts. The hosts themselves are not able to understand the values stored in their databases because each value looks like random noise owing to secret sharing. However, it is not trivial to perform computations on secret shared values as special secure multi-party computation protocols are required. The secure computation protocols used by the hosts preserve the privacy of the data during computation. The genotype data remain secure as long as the hosts do not share their databases of shares with each other. Figure 1 shows how secure multi-party computation works with secret sharing.

Secure computation can be used to perform most data processing operations. However, current solutions have some important differences compared with standard programming: (i) floating-point operations are significantly slower; (ii) comparison operations are slower than multiplication and addition; (iii) parallel execution of several operations is faster than sequential execution. Further details can be found in the Supplementary Data and in the articles by Bogdanov *et al.* (2008); Damgård *et al.* (2009) and Geisler (2010).

### 3.3 Secure storage of genotype data

Allele-level descriptions of genotypes are commonly stored as sequences of pairs where each pair is encoded as AA, AB, BB or NN, corresponding to a specific SNP. In GWAS, such data are converted into contingency tables as described in Tables 1 and 2 depending on the analysis method being used.

This kind of counting, however, requires the use of string comparison operations that we would like to avoid, as they tend to be relatively slow in the case of share computing. Therefore, we represent each SNP as a pair of integers 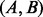, where 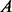 counts the occurrences of the first allele and 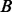 the occurrences of the second allele in that SNP. That is, pairs AA, AB, BB, NN are encoded as (2,0), (1,1), (0,2), (0,0), respectively.

During the data collection phase, data donors, wetlabs or gene banks must first convert genotypes into the form described above and then secret share them between data hosts. As a result, we get a secret shared database where each column corresponds to an individual donor and there are two rows for each SNP (one for 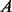 and one for 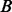). For each donor, we also store an ID value that uniquely identifies them.

### 3.4 Secure formation of case–control groups

To hide the identities of case and control group members, secret shared index vectors are used to specify the groups. An index vector 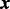 is a zero-one vector, where *x_i_* corresponds to the *i*-th column in the database and 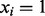 if and only if the *i*-th person is a member of the group that vector represents.

The two principal ways of how to construct case–control groups can be seen in Figure 2 Panel C. In the first scenario, analysts have direct access to clinical data for a set of donors and, thus, are able to select case and control groups and construct index vectors. In the second scenario, phenotype data are also stored in secret shared form. In this case, there exists a secret shared database of phenotype attributes that consists of boolean attributes (e.g. has diabetes) and integers (e.g. age, blood pressure, height). To construct case and control groups, the analyst has to specify a logical expression based on which the hosts perform the necessary comparisons and output the two index vectors in secret shared form. For simple inclusion criteria, such as the one we used for experiments 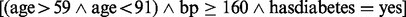, the corresponding secure comparison protocol is rather efficient.

### 3.5 Secure assembly of contingency groups

Let 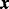 and 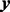 be index vectors for case and control groups, respectively. Let 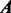 and 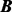 be database columns for a particular SNP. Then the allele counts needed for tests (1) and (2) can be expressed as
(5)
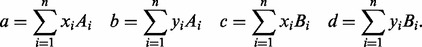

Data hosts can securely compute shares of 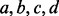 for each SNP.

For the second contingency table, 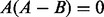 for allele combinations AB, BB and NN. Similarly, 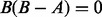 for AA, AB and NN and 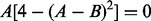 for AA, BB and NN. We can express counts as
(6)
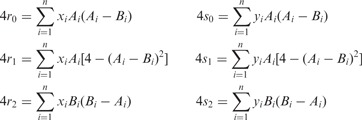

For TDT, we need to construct an index vector 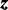 for detecting heterozygous parents, i.e. 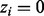 if at least one parent is homozygous and 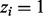 otherwise. As 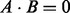 for homozygous allele combinations, we compute
(7)
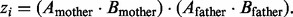

To get shares of *u* and *v*, we can combine *z* with counts of AA and BB.

### 3.6 Secure statistical testing

To determine whether a SNP is significant, one must check whether a *P*-value that is associated with a test statistic is below a pre-described significance level. Let *α* be the desired significance level and let 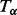 be such that 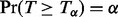 when *T* is distributed according to 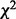 distribution with one degree of freedom. A SNP is significant only if the corresponding test statistic is 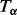.

Direct evaluation of formulae (1)–(4) requires floating-point arithmetic, which we would like to avoid. Hence, we must rewrite the equation 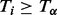 in terms of integer operations. Let 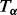 be represented as a fraction *p*/*q* and the test statistic as a fraction *m*/*n*. Then the condition 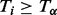 is equivalent to the condition 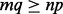. Both sides of this inequality can be securely computed and the inequality can be evaluated by using secure comparison operation, after which we can publish the comparison results to find out which SNPs are significant.

The significance level must be determined considering the multiple testing issue. The simplest way is to use Bonferroni correction, which is a conservative measure. It is also possible to perform privacy-preserving FDR correction. We estimate that the secure version of the standard FDR procedure takes about 10 min to complete for 262 264 SNPs. However, there are alternatives to the original algorithm that are significantly faster; see the Supplementary Data for further details.

## 4 RESULTS

To demonstrate the feasibility of our approach, we used the Sharemind multi-party computation platform (Bogdanov *et al.*, 2008) to implement core algorithms for GWAS. Our choice was mainly motivated by the efficiency and ease of use of the Sharemind platform. Alternative platforms [Viff (Damgård *et al.*, 2009) and FairplayMP (Ben-David *et al.*, 2008)] should give similar results.

We used 270 genotypes from the HapMap project (International HapMap Consortium, 2003) measured with the Affymetrix Mapping 500K Array as the main data source. In each experiment, we divided the data randomly into case and control groups and performed genome-wide search for highly differentiated SNPs. For that we used cryptographically secure counterparts of standard statistical tests used in GWAS: two 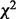 tests for independence (Visscher and Hellard, 2003), Cochran–Armitage test for trend (Sasieni, 1997) and TDT (Spielman *et al.*, 1993). As our algorithms return exactly the same outputs as original algorithms, we report only performance results for various sub-tasks. To show the variability of running times, we report the mean and standard deviation of four independent runs.

Each of the donors has 262 264 measured SNPs. First, we ran the algorithm on the data of 270 donors, and then we went on to test the data of 540, 810 and 1080 donors. We performed the experiments on three servers running Sharemind. Each server was an off-the-shelf server-grade machine with 48 GB RAM of which less was used, twelve 2.93 GHz Intel Xeon (Westmere) cores of which two were used and a 1 Gb/s local area network (LAN) connection. At the moment, the network connection is the bottleneck in terms of algorithm running time; however, Sharemind has been successfully used in real applications (Bogdanov *et al.*, 2012).

The time spent on data acquisition and secure storage does not depend on the statistical test used later on. It depends only on the number of SNPs and the number of gene donors. The average time it takes to encode and share the SNPs for the described case can be seen in Table 3. Note that secret sharing and uploading data are done only once for each dataset; hence, this is a single-time cost.

**Table 3.** Performance results for data upload and filtering

Number of donors	Upload	Filtering
270 donors	12.0 min	0.51 s
540 donors	17.3 min	0.59 s
810 donors	23.2 min	0.63 s
1080 donors	29.4 min	0.68 s

The time needed to form case–control groups depends on the application scenario. When the analyst has direct access to phenotype data and can form case and control groups by herself/himself, then there is no computational overhead. In more involved cases, the case and control groups must be constructed based on secret shared phenotype attributes. In this case, the overhead depends on the complexity of inclusion criteria for case and control groups. Filtering results presented in Table 3 show that formation of such groups can be done in seconds for typical inclusion criteria that consist of simple comparison operations mixed with logical conjunctives.

The time needed to perform the statistical test depends on the test, but in all cases it can be broken down into counting allele frequencies and evaluating test statistic. As Tables 4 and 5 clearly show, the main performance bottleneck is frequency counting, which scales linearly w.r.t. the total number of SNP measurements. As our encoding is optimized for 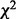 test, a better encoding will enhance the performance of the Cochran–Armitage tests but not beyond 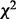 results. The total duration of the analysis is the sum of the frequency analysis and evaluation, as the other parts have a negligible duration.

**Table 4.** Performance results for three different frequency analyses

Number of donors	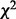 tests	Cochran–Armitage	TDT
270 donors	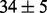 min	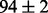 min	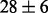 min
540 donors	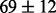 min	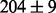 min	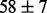 min
810 donors	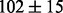 min	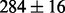 min	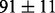 min
1080 donors	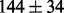 min	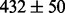 min	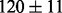 min
1080 donors (non-secure)	14 s	35 s	11 s

**Table 5.** Performance results for four test evaluation methods

Number of donors	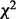 test 1	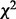 test 2	Cochran– Armitage	TDT
270 donors	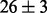 s	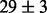 s	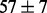 s	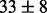 s
540 donors	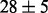 s	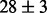 s	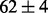 s	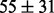 s
810 donors	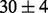 s	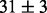 s	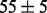 s	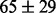 s
1080 donors	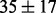 s	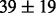 s	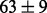 s	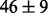 s
1080 donors (non-secure)	21 ms	20 ms	49 ms	11 ms

The presented results clearly show that cryptographically secure evaluation of statistical tests on genome-wide scale is practically feasible. The expected running time is hours instead of a few minutes when computed non-securely. However, the latter is not a significant slowdown compared with the time needed to acquire the data if the secure analysis method is not used.

## 5 DISCUSSION

Although the results prove the practical feasibility of cryptographically secure GWAS, the solution is also notably more resource demanding than the alternatives. We have to analyze further whether potential benefits outweigh costs. We consider three potential application scenarios and contrast our approach with the alternatives.

### 5.1 Collaboration between hospitals and biobanks

By now many countries have national or state-funded biobanks. There are more than 40 state-governed biobanks in Europe (Zika *et al.*, 2010) and many others in Asia and America (Swede *et al.*, 2007). Thus, there is a huge potential for collaborative studies between biobanks, hospitals and pharmaceutical companies. For example, if a clinical study indicates that a treatment is ineffective for a certain group of people, a genome-wide association study can indicate whether a particular set of SNPs can be used to predict efficacy of a treatment. In particular, GWAS have shown that certain SNP mutations influence efficacy of treatments for asthma, inflammatory bowel disease, coronary heart disease and cancer (Grant and Hakonarson, 2007).

Privacy issues are the major obstacle in such studies: neither biobanks nor research institutions can give out data without explicit consent from the patients or explicit decision by a relevant ethics committee. In such scenarios, secure GWAS can be used as a pilot study for assessing potential benefits of combined studies. In particular, there is no reason to merge the data for further analysis if a secure GWAS reveals no differentially expressed SNPs. Because such a study can be conducted in a few hours, it can significantly speed up pharmacogenetic studies and the results can be used by ethics committees to make more seasoned decisions.

### 5.2 Servicing study data without privacy breaches

In many state-funded studies, collected data must be made accessible for public use by submitting them to a central repository. The NIH example shows a direct publication of GWAS data may cause unintended consequences even if the data are presented to the public in aggregated form (Couzin, 2008). One potential solution is to set up an online web service for conducting GWAS: a researcher just posts inclusion criteria for case and control groups and all computations are done by the host. In most cases, such a solution is adequate without cryptographic countermeasures. Privacy issues emerge only if researchers want to pool together data from several different repositories to detect weak associations or the inclusion criteria must remain private. In these cases, secure GWAS methodology can be applied by combining methods for international consortium studies with phenotype-based filtering (see Fig. 2).

### 5.3 Faster and more secure consortium studies

One of the main hurdles in GWAS is the sample size. For rare diseases, there are not enough genotyped patients to form a big enough case group. Also, over- and under-representation of sub-populations can cause spurious associations. Larger studies involving several international biobanks can diminish the impact of such problems.

Two competing alternatives to our solution in this setting are federated database systems with additional security mechanisms and hierarchical aggregation of the data.

A federated database system offers middleware that automatically binds together several sources and allows users to make various queries without knowing how the data are organized. As such, a federated database system does not solve privacy issues. Hence, one needs an honest broker—a dedicated server with strengthened security measures that assembles the data and processes all queries (Boyd *et al.*, 2007). The latter introduces a single point of failure—nothing can be done if the security of the honest broker is breached. Also, as the data are sent directly to the honest broker, all biobanks must have explicit clearance for releasing the data.

Hierarchical data aggregation is applicable when results of individual studies can be combined with some meta-analysis technique (e.g. Wolfson *et al.*, 2010). In such cases, the honest broker must access only aggregated summaries of individual data sources to produce the desired result. Consequently, we get stronger privacy guarantees, as the broker receives only a limited amount of information. However, unexpected privacy breaches can still occur because aggregation methods provide no explicit security guarantees and it is extremely difficult to assess how much information is leaked through summaries. Also, the approach cannot be used when members of case and control groups must be kept secret.

In a nutshell, while the alternatives are faster than our solution, they are also much more vulnerable to various attacks, and thus, privacy concerns can prevent their usage or considerably delay the initial setup time. Moreover, hierarchical data aggregation techniques can be combined with our solution. Namely, our solution can be used to replace the honest broker—biobanks secret share the aggregated results, and thus, fewer operations must be done on shares. For the analysis part of GWAS, the resulting hybrid algorithm will only take the time given in Table 5 plus a little overhead, making the computation time ∼30 s to 1 min as the filtering of case and control groups and computation of contingency tables are done locally.

### 5.4 Long-term security in a personal genomic project

The rapid decrease of genotyping costs and moderate success in genetic diagnostics have sparkled interest in personal genomics. Companies, such as 23andMe, deCODEme and Navigenics, offer personalized genotyping services. Although participants have a right to withdraw their data at any moment, this right is enforced only by physical and organizational methods. As a consequence, a single successful outsider or insider attack can obsolete all privacy guarantees. Numerous data leakages in other areas have shown that this is an irreversible procedure. Once data have leaked, there is no way to recall them. On a shorter timescale, such events are highly improbable. However, such projects need privacy guarantees that last more than 100 years to protect participants and their offspring. In such settings, distributed storage based on secret sharing is one of the best cryptographic alternatives, as a successful breach of security of a single facility yields no information and it is possible to recover from such events.

*Funding*: The research was supported by the European Regional Development Fund through Estonian Centre of Excellence in Computer Science and Software Technology and Applications Competence Center.

*Conflict of Interest*: none declared.

## Supplementary Material

Supplementary Data
