# Supplementary material for: A new way to protect privacy in large-scale genome-wide association studies
Source: Bioinformatics. 2013 Feb 14;29(7):886–93. doi: 10.1093/bioinformatics/btt066 (PMC3605601; doi:10.1093/bioinformatics/btt066)
Supplement: Supplementary Data [file supp_29_7_886__index.html]

A new way to protect privacy in large-scale genome-wide association studies — A new way to protect privacy in large-scale genome-wide association studies — Supplementary Data 

# A new way to protect privacy in large-scale genome-wide association studies

## Supplementary Data

files

**Files in this Data Supplement:**

- Supplementary Data - pdf file
