# Supplementary material for: A new way to protect privacy in large-scale genome-wide association studies
Source: Bioinformatics. 2013 Feb 14;29(7):886–93. doi: 10.1093/bioinformatics/btt066 (PMC3605601; doi:10.1093/bioinformatics/btt066)
Supplement: Supplementary Data [file supp_btt066_supplementary_pdf.pdf]

How to mine genes without violating privacy: Technical Supplementary

Liina Kamm, Dan Bogdanov, Sven Laur, Jaak Vilo

Abstract

This supplementary highlights some technical issues we decided to omit from the article because of the higher level of technical detail. First of all, it describes security and performance issues related to secure multi-party computations. Second, it provides formal reasoning why the implementation of all algorithms leaks no information beyond the intended outputs. Third, we fully describe all secure genome-wide association study algorithms that were used to achieve our experiment results.

Contents

1 Practical Details of Privacy-Preserving Data Mining **3**
1.1 Main Properties of the Sharemind Framework . . . 3
1.2 Developer Tools in the Sharemind Framework . . . 5

2 Algorithm Notation **5**

3 Calculating Frequencies for Cases and Controls **7**
3.1 χ^2 Algorithms . . . 7
3.2 Cochran-Armitage Test . . . 7
3.3 Transmission Disequilibrium Test . . . 8
3.4 Security analysis . . . 10

4 Privacy-Preserving Testing Algorithms **10**
4.1 General Algorithm Information . . . 10
4.2 Standard χ^2 Test . . . 10
4.3 Equiproportionality of Allele A in Both Groups . . . 11
4.4 Cochran-Armitage Test . . . 11
4.5 Transmission Disequilibrium Test . . . 12
4.6 Security analysis . . . 13
4.7 Multiple testing and FDR correction . . . 13
4.8 Optimisations for the FDR correction procedure . . . 15

5 Privacy-Preserving Methods for Quality Control **15**
5.1 Algorithms for Monitoring Data Quality . . . 15
5.2 Examples of Outlier Detection and Filtering Algorithms . . . 16

6 Performance evaluation **17**

1 Practical Details of Privacy-Preserving Data Mining

Privacy-preserving data mining is a cover term for a set of techniques that allow sensitive data to be processed with minimal or no leaks. In our work, we use secure multi-party computation which allows us to securely evaluate a function among several parties so that none of them learns anything except for their own inputs and the final outputs. More precisely, we chose the Sharemind secure computation framework [BLW08] as a concrete implementation platform.

1.1 Main Properties of the Sharemind Framework

All computations in the Sharemind platform are based on additive secret of 32-bit and 64-bit integers. Hence, data donors can distribute their secret values among the hosts so that none of the hosts nor a subset of them can recover anything about the inputs unless all of the hosts pool their databases. More formally, a secret sharing scheme splits a secret value into several pieces (called *shares*) so that it is infeasible to reconstruct the original value without access to a predetermined number of shares. In the case of additive secret sharing, we need all shares to reconstruct the secret. The domain of secret sharing determines what type of data can be stored and which operations can be performed on the data without revealing secrets. With Sharemind we can perform secure arithmetic with 32-bit and 64-bit unsigned integers.

The general idea of computations on Sharemind are shown in Figure 1. Input values are secret-shared at the source and collected by three servers. The servers run secure multi-party computation protocols (distributed computations), which process input shares and output results in the form of new shares. These protocols guarantee that nothing is leaked about the inputs and outputs. Thus, data hosts learn only the types of computation steps they perform and nothing more. In the end of the computation, the shares of the end result are published to the parties who are interested in the result, who can reconstruct it since they have all the shares.

As the Sharemind platform naturally supports basic integer operations, we converted all algorithms into the form, where they are specified in terms of addition, multiplication and comparison operations. The latter makes our algorithms relatively fast since secure operations for fixed and floating point arithmetic are significantly slower.

Another advantage of the Sharemind platform is the *universal composability* of all operations. Note that not all secure multi-party computation protocols preserve security guarantees if they are executed together with other protocols—sometimes it is possible to break the confidentiality of protocols by gathering information from several parallel protocol runs.

Parallel execution of several multi-party protocols is guaranteed to preserve security if all protocols are universally composable. As all Sharemind protocols have been shown to be universally composable [BLW08, BNTW12], we can execute several operations in parallel. This can give us significant speed-ups, as the price of sending a value over the network in Sharemind is similar for single values and vectors of small values. To use this speedup to one's advantage, it is possible to instruct Sharemind to perform componentwise operations on entire vectors and matrices in addition to processing single values. These parallel operations are significantly more efficient than single operations because several values can be packed into fewer network messages.

For an example of the gains, see Figures 2. The horizontal axis shows input vector sizes from a single value to one million values on a logarithmic scale and the vertical axis shows the running time of the multiplication in milliseconds. Two lines have been fit on the average running times—the horizontal line shows that processing a single value and processing one thousand values takes nearly the same amount of time. The second line shows a steeper increase in running times. From our benchmarking experiments we have determined that the running time becomes nearly linear in the size of the vector once the network bandwidth is used to its limits.

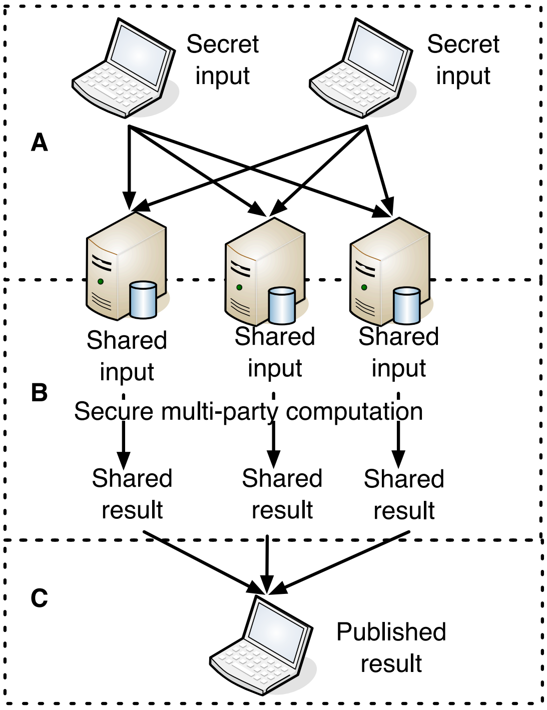

Figure 1: Processing secure computation using secret sharing.

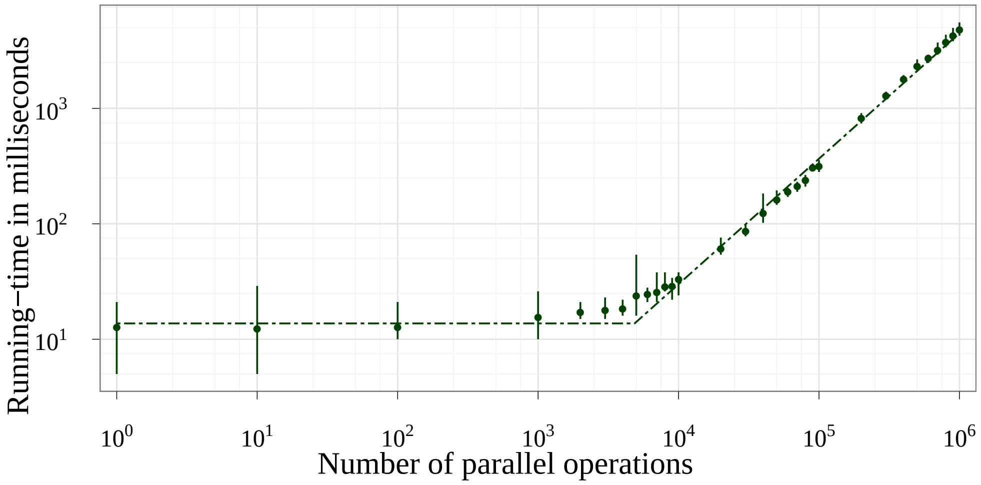

Figure 2: Effect of vectorization on secure multiplication running time.

The efficiency of parallel operations motivates us to design the secure computation algorithms in a manner that stresses large vector operations. However, the latter is not the entire truth. Working with large vectors also increases the memory footprint. Controlling memory usage is significant in the case of genome-wide association studies because of the data size. Moreover, performing more operations decreases the average cost of an individual operations only up to a certain number of operations, see Figure 2. Therefore, it is reasonable to do operations in batches, cutting larger vectors into smaller pieces.

Although all our implementations use explicit batching to reduce the memory footprint, we decided to omit these details. Firstly, it complicates the overall presentation of algorithms. Secondly, it is straightforward to add batching to algorithms by dividing the SNP data into parts and processing these in succession.

1.2 Developer Tools in the Sharemind Framework

SHAREMIND is a general secure multi-party computation system in the sense that it is not limited to solving a single problem with a dedicated protocol. The universally composable protocol suite enables the virtual machine inspired design of SHAREMIND and allows us to program this machine as we would program a standard computer. SHAREMIND natively executes an assembly language that has instructions for both public and private operations. Public operations are used for non-sensitive data like loop invariants, data structure sizes and conditional expressions. SHAREMIND does not hide the flow of the code being executed and instead provides means for hiding branching decisions automatically. All branching decisions in SHAREMIND are based on public values.

The SHAREMIND framework uses a high-level programming language called SECREC [Jag10]. The main feature of SECREC is a type system that separates public and private data. During execution, private data is processed using secure multi-party computation and public data is processed as usual. Interaction between public and private data is controlled as follows:

- public values can automatically become private and
- private values can become public only through the `declassify`() operator.

The use of a dedicated declassification operator explicitly shows the places in the algorithm where private data is made public (public values are reconstructed from shares). To show that an algorithm does not leak sensitive data, the developer needs to show that none of the declassification calls leak information about the sensitive inputs.

SECREC also includes support for vectors and matrices of both private and public data. SECREC allows vectors and matrices of the same size to be used in componentwise operations and also aggregation operations like computing a sum of all the values in a vector. It is possible to work with SHAREMIND's secure database from within SECREC code.

SECREC enables another process in the development of secure applications—since a SECREC program specifies, what computations a SHAREMIND node can perform and what it can publish, SECREC code can be audited before deployment by each secure multi-party computation host.

2 Algorithm Notation

Let $\boldsymbol{x}$ denote a vector and let $\boldsymbol{x} + \boldsymbol{y}$ denote the componentwise addition of vectors and $\boldsymbol{x} \odot \boldsymbol{y}$ the componentwise multiplication of vectors. When a scalar value is used in the place of a vector, it is automatically extended to a vector of the same size as the other vector operand. Vector operation precedence is the same as scalar operation precedence—multiplications are performed

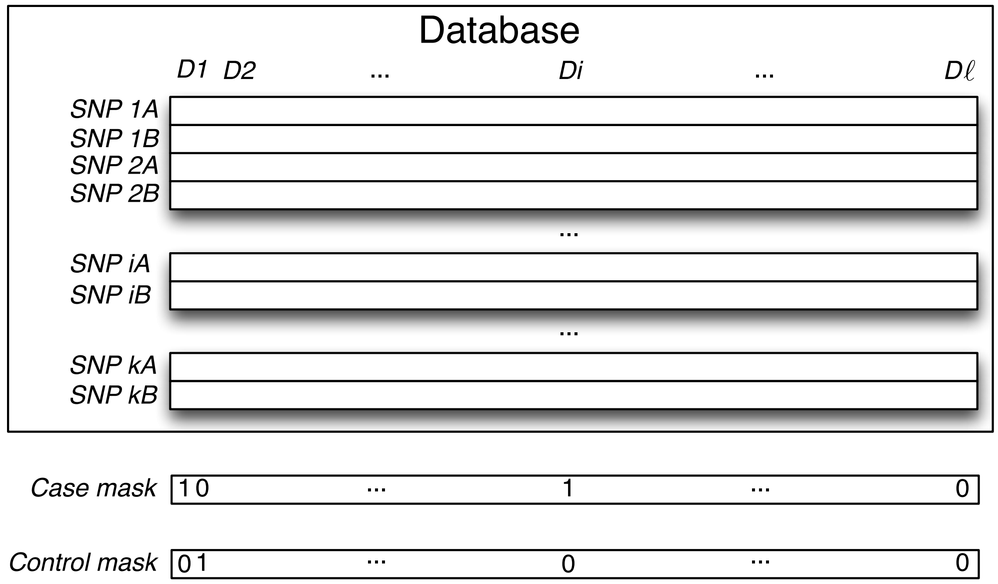

Figure 3: Schematic visualisation of database layout together with mask vectors

before additions and so on. By $\boldsymbol{x}[i]$, we denote the i-th element of $\boldsymbol{x}$. In addition, let $\mathcal{SDB}$ denote a matrix (SNP database) and let $\mathcal{SDB}[i, *]$ denote the i-th row of $\mathcal{SDB}$.

All privacy-preserving algorithms presented below use private values stored as shares and public values available for all secure multi-party computation hosts. To make a clear distinction, we surround private variables with double brackets and write public variables as usual. For example, $[\![x]\!]$ denotes that variable x is secret shared and $[\![z]\!] \leftarrow [\![x]\!] \odot [\![y]\!]$ means that the shares of $z = x \cdot y$ are securely computed from the shares of x and y. The same notational convention applies to vectors and matrices, e.g $[\![\boldsymbol{x}]\!] + [\![\boldsymbol{y}]\!]$ denotes component-wise addition of shared vectors.

As in the main article, we assume that four possible states of a SNP are reduced to two alleles: a dominant base pair and a mutation. The resulting data is stored in the shared genotype database $[\![\mathcal{SDB}]\!]$ with $2k$ rows and ℓ columns where k is the number of SNPs and ℓ is the number of donors. Rows of $[\![\mathcal{SDB}]\!]$ come in pairs—the first row in every pair contains the count of allele A in the SNP and the second row the count of allele B. Each column represents one donor.

We represent case and control groups as shared zero-one index vectors (*masks*). Let $[\![\boldsymbol{mask}]\!]$ be an index vector for the case group. Then the ith donor belongs to the case group if and only if $\boldsymbol{mask}[i] = 1$. See Figure 3 for an illustration of the database layout and mask vectors. Algorithm 1 shows how to securely compose the mask based on range filters. The first input of the algorithm is a vector $[\![\boldsymbol{attribute}]\!]$ with length ℓ (the attribute value for each donor). The second and third inputs are the filter's lower and upper bounds respectively. Since the Sharemind implementations of smaller and greater than predicates always return zero or one, the mask $\boldsymbol{mask}[i] = 1$ if and only if $minimum \leq \boldsymbol{attribute}[i] \leq maximum$.

Algorithm 1: Calculating a range mask based on range limits and an attribute

```
RangeFilter([[attribute]], [[minimum]], [[maximum]])
begin
    [[ltemask]] ← [[attribute]] ≤ [[maximum]];
    [[gtemask]] ← [[attribute]] ≥ [[minimum]];
    return [[ltemask]] ⊙ [[gtemask]];
```

Algorithm 2: Counting a specified allele in a specified population

```
GetAlleleCountChiSq([[SDB]], k, allele, [[mask]])
begin
    for i ∈ {1, ..., k} do
        [[alleleData]] ← [[SDB]][2(i − 1) + allele, ∗];
        [[maskedAllele]] ← [[alleleData]] ⊙ [[mask]];
        [[support]][i] ← ∑ [[maskedAllele]]
    return [[support]];
```

3 Calculating Frequencies for Cases and Controls

3.1 χ^2 Algorithms

Let $\boldsymbol{x}$ and $\boldsymbol{y}$ be masks for case and control groups respectively. Let A and B be the database columns for a particular SNP. Then the allele counts can be expressed as

$$\begin{aligned} a &= \sum_{i=1}^{n} x_i A_i & c &= \sum_{i=1}^{n} x_i B_i \\ b &= \sum_{i=1}^{n} y_i A_i & d &= \sum_{i=1}^{n} y_i B_i \end{aligned} \tag{1}$$

Based on the equations (1), Algorithm 2 calculates for each SNP the number of alleles (A or B) in a given group (cases or controls). The first input to Algorithm 2 is the shared genotype database $[\![\mathcal{SDB}]\!]$. The second input k gives the number of rows in the database and the third input $allele \in \{1, 2\}$ denotes the allele that we are aggregating where 1 stands for allele A and 2 stands for allele B. The final input, is the mask $[\![\boldsymbol{mask}]\!]$.

3.2 Cochran-Armitage Test

To compile the contingency table seen in Table 1 for the Cochran-Armitage test, note that $A_i(A_i - B_i) = 0$ if the ith donor has allele combinations AB, BB and NN for the particular SNP. Similarly, $B_i(B_i - A_i) = 0$ for allele combinations AA, AB and NN and $A_i(4 - (A_i - B_i)^2) = 0$

Table 1: Contingency table for the Cochran-Armitage test for trend

	**Allele AA**	**Allele AB**	**Allele BB**	**Total**
**Cases**	r_0	r_1	r_2	m_1
**Controls**	s_0	s_1	s_2	m_2
**Total**	n_0	n_1	n_2	N

Algorithm 3: Determining the genotype distribution for a group specified with $\boldsymbol{mask}$

```
GetGenotypeDistr([[SDB]], k, [[mask]])
begin
    for i ∈ {1, ..., k} do
        [[maskedA]] ← [[SDB]][2(i − 1) + 1, ∗ ] ⊙ [[mask]];
        [[maskedB]] ← [[SDB]][2(i − 1) + 2, ∗ ] ⊙ [[mask]];
        // Sum of donors with AA
        [[sumAA]][i] ← ∑([[maskedA]] ⊙ ([[maskedA]] − [[maskedB]]));
        // Sum of donors with AB
        [[difAB]] ← [[maskedA]] − [[maskedB]];
        [[sumAB]][i] ← ∑([[maskedA]] ⊙ (4 − [[difAB]] ⊙ [[difAB]]));
        // Sum of donors with BB
        [[sumBB]][i] ← ∑([[maskedB]] ⊙ ([[maskedB]] − [[maskedA]]));
    return ([[sumAA]], [[sumAB]], [[sumBB]]);
```

for allele combinations AA, BB and NN. Hence, we can express the counts with factor 4 as

$$
\begin{aligned}
4r_0 &= \sum_{i=1}^{n} x_i A_i (A_i - B_i) & 4s_0 &= \sum_{i=1}^{n} y_i A_i (A_i - B_i) \\
4r_1 &= \sum_{i=1}^{n} x_i A_i (4 - (A_i - B_i)^2) & 4s_1 &= \sum_{i=1}^{n} y_i A_i (4 - (A_i - B_i)^2) \\
4r_2 &= \sum_{i=1}^{n} x_i B_i (B_i - A_i) & 4s_2 &= \sum_{i=1}^{n} y_i B_i (B_i - A_i)
\end{aligned}
\tag{2}
$$

Thus, the contingency table is also easily computable.

Based on the equations (2), Algorithm 3 calculates the frequencies of allele pairs AA, AB and BB for each SNP. The first input to Algorithm 3 is the shared genotype database $[\![\mathcal{SDB}]\!]$ with the number of rows given in the second input k. The third input is the group mask $[\![\boldsymbol{mask}]\!]$.

It is important to note, that according to the equations (2), all of the calculated counts are fourfold. This will be taken into account in the algorithms where the test statistics are calculated.

3.3 Transmission Disequilibrium Test

Transmission disequilibrium test (TDT) is applicable only if the data consist of parent-child trios. The test measures whether one homozygous genotype is more over-represented than the other among affected children with heterozygous parents. For this we need to construct an index vector

Algorithm 4: Determining the allele counts of homozygous children

Database layout: Parent-child trios are stored as consecutive column triples.

```
GetHomozygousChildCounts([[SDB]], k, ℓ, [[childMask]])
begin
    for i ∈ {1, ..., k} do
        [[alleleAData]] ← [[SDB]][2(i − 1) + 1, ∗];
        [[alleleBData]] ← [[SDB]][2(i − 1) + 2, ∗];
        //Extract mothers' data from the database;
        P_1 = {3(j − 1) + 1 : j = 1, ..., ℓ/3};
        [[p1AlleleA]] ← [[alleleAData]][P_1];
        [[p1AlleleB]] ← [[alleleBData]][P_1];
        //Extract fathers' data from the database;
        P_2 = {3(j − 1) + 2 : j = 1, ..., ℓ/3};
        [[p2AlleleA]] ← [[alleleAData]][P_2];
        [[p2AlleleB]] ← [[alleleBData]][P_2];
        //Extract children's data from the database;
        C = {3(j − 1) + 3 : j = 1, ..., ℓ/3};
        [[childA]] ← [[alleleAData]][C];
        [[childB]] ← [[alleleBData]][C];
        //Construct masks for the heterozygous parents
        [[p1Mask]] ← [[p1AlleleA]] ⊙ [[p1AlleleB]];
        [[p2Mask]] ← [[p2AlleleA]] ⊙ [[p2AlleleB]];
        //Construct the mask for the selected homozygous children
        [[mask]] ← [[p1Mask]] ⊙ [[p2Mask]] ⊙ [[childMask]];
        // Find the fourfold counts of AA and AB for the selected homozygous children
        [[sumAA]][i] ← ∑([[mask]] ⊙ [[childA]] ⊙ ([[childA]] − [[childB]]));
        [[sumBB]][i] ← ∑([[mask]] ⊙ [[childB]] ⊙ ([[childB]] − [[childA]]));
    return ([[sumAA]], [[sumBB]]);
```

for detecting heterozygous parents. Note that $A_i \cdot B_i = 0$ for homozygous allele combinations and, thus, we can construct an index vector

$$\boldsymbol{z} = (A_{mother} \cdot B_{mother}) \cdot (A_{father} \cdot B_{father}) \tag{3}$$

that is 1 if both parents are heterozygous and 0 otherwise.

Based on the equations (2) and (3), Algorithm 4 computes the fourfold counts of genotypes AA and BB of children. This will, again, be taken into account in the next step. The first input to Algorithm 4 is the shared genotype database $[\![\mathcal{SDB}]\!]$ with k rows and ℓ columns. The fourth input is a zero-one mask $[\![\boldsymbol{childMask}]\!]$ with length $\ell/3$, that contains 1 if the child has the disease in question. This mask is different from the ones used in the previous algorithms as it is only applied to 1/3 of the donors, namely the children. For this test, we assume that parent-child trios are stored as consecutive column triples, where the first column corresponds to the mother, the second to the father and the final to the child. This assumption is just for notational convenience. One can easily modify the algorithm for arbitrary donor layout, as long as the information about which columns correspond to each mother-father-child trio is public.

3.4 Security analysis

The SHAREMIND framework guarantees that nothing about the input and outputs are leaked when the secure computations use input shares to produce output shares [BLW08]. All algorithms presented so far declassify no values and therefore all these algorithms are secure. More formally, note that the flow of the protocol execution is independent from the genotype data and masks used to select donors. Hence, the corresponding simulation strategy in the formal security proof is straightforward: use canonical simulators for each private operation in the algorithm specification.

4 Privacy-Preserving Testing Algorithms

4.1 General Algorithm Information

All four algorithms given in this section calculate and publish a zero-one vector $\boldsymbol{significance}$ that is set to 1 if the SNP is significant according to the test statistic in question.

The arguments for all functions are the same in general. As the first input, the algorithms are given the shared genotype database $[\![\mathcal{SDB}]\!]$ with $2k$ rows and ℓ columns where k is the number of SNPs and ℓ is the number of donors. The third and fourth input $[\![\boldsymbol{casemask}]\!]$ and $[\![\boldsymbol{ctrlmask}]\!]$ are the shared case and control masks that have the value 1 for each donor that belongs into the respective group, and 0 otherwise. These vectors are either given explicitly by the data analyst or computed securely form secret shared phenotype attributes with algorithms similar to Algorithm 1.

As mentioned in Section 1, floating-point arithmetic is very slow and its implementation is too imprecise for our needs. In the algorithms, therefore, instead of division, we rewrite the equation $T \geq T_\alpha$ in terms of integer operations. Let T_α be represented as a fraction $\frac{p}{q}$ and the test statistic as a fraction $\frac{x}{y}$. Then the condition $T \geq T_\alpha$ is equivalent to the condition $xq \geq yp$. The last two inputs p and q of the algorithms are the dividend and divisor that make up the user specified threshold $\frac{p}{q}$ to which the test statistic is compared.

4.2 Standard χ^2 Test

Let a, b, c, and d denote the count of observed alleles A and B in cases and controls, as specified in the main body of the article. The test statistic for the standard χ^2-test can be expressed as

$$T_1 = \frac{(a+b+c+d)(bc-ad)^2}{(a+b)(a+c)(b+d)(c+d)} \ . \tag{4}$$

Algorithm 5 uses these four variables to calculate the test statistic T_1 based on equation (4). The data for the contingency table is obtained by using Algorithm 2.

Using the reasoning in Subsection 4.1 to avoid floating-point operations, equation (4) gives us a test

$$q(a+b+c+d)(bc-ad)^2 \geq p(a+b)(a+c)(b+d)(c+d) \tag{5}$$

where the threshold $T_\alpha = p/q$ can be determined by computing upper α quantiles of the χ^2-distribution with one degree of freedom. We discuss how to determine appropriate significance level α in Subsection 4.7.

Algorithm 5: Standard χ^2 Test

```
StandardChiSqTest([[SDB]], k, [[casemask]], [[ctrlmask]], p, q)
begin
    [[a]] ← GetAlleleCountChiSq([[SDB]], k, 1, [[casemask]]);
    [[b]] ← GetAlleleCountChiSq([[SDB]], k, 1, [[ctrlmask]]);
    [[c]] ← GetAlleleCountChiSq([[SDB]], k, 2, [[casemask]]);
    [[d]] ← GetAlleleCountChiSq([[SDB]], k, 2, [[ctrlmask]]);
    [[sumcases]] ← [[a]] + [[c]];
    [[sumctrls]] ← [[b]] + [[d]];
    [[x]] ← [[b]] ⊙ [[c]] − [[a]] ⊙ [[d]];
    [[x]] ← [[x]] ⊙ [[x]] ⊙ ([[sumcases]] + [[sumctrls]]);
    [[y]] ← ([[a]] + [[b]]) ⊙ ([[c]] + [[d]]) ⊙ [[sumcases]] ⊙ [[sumctrls]];
    [[significance]] ← q ⊙ [[x]] ≥ p ⊙ [[y]];
    return declassify([[significance]]);
```

Algorithm 6: Test for equiproportionality of allele A in both groups

```
AlleleAEquipropTest([[SDB]], k, [[casemask]], [[ctrlmask]], p, q)
begin
    [[a]] ← GetAlleleCountChiSq([[SDB]], k, 1, [[casemask]]);
    [[b]] ← GetAlleleCountChiSq([[SDB]], k, 1, [[ctrlmask]]);
    [[c]] ← GetAlleleCountChiSq([[SDB]], k, 2, [[casemask]]);
    [[d]] ← GetAlleleCountChiSq([[SDB]], k, 2, [[ctrlmask]]);
    [[sumcases]] ← [[a]] + [[c]];
    [[sumctrls]] ← [[b]] + [[d]];
    [[x]] ← [[b]] ⊙ [[c]] − [[a]] ⊙ [[d]];
    [[x]] ← 2 ⊙ [[sumctrls]] ⊙ [[x]] ⊙ [[x]];
    [[y]] ← [[b]] ⊙ [[sumcases]] ⊙ [[sumcases]] ⊙ [[d]];
    [[significance]] ← q ⊙ [[x]] ≥ p ⊙ [[y]];
    return declassify([[significance]]);
```

4.3 Equiproportionality of Allele A in Both Groups

To determine whether allele A is equiproportional in both the case and control group, we use the same counts a, b, c and d as for the standard χ^2 test. Thus, the initial part of Algorithm 6 coincides with Algortihm 5. As the test statistic for determining the equiproportionality is

$$T_2 = \frac{2(b+d)(bc-ad)^2}{b(a+c)^2 d} \quad , \tag{6}$$

Algorithm 6 uses the condition

$$2q(b+d)(bc-ad)^2 \geq pb(a+c)^2 d$$

to sieve out significant cases.

4.4 Cochran-Armitage Test

To calculate the test statistic for the Cochran-Armitage test for trend, we need compute all counts in Table 1. The data for the contingency table is obtained by using Algorithm 3.

Algorithm 7: Cochran-Armitage test for trend

```
CochranArmitageTest([[SDB]], k, [[casemask]], [[ctrlmask]], α0, α1, α2, p, q)
begin
    ([[r0]], [[r1]], [[r2]]) ← GetGenotypeDistr([[SDB]], k, [[casemask]]);
    ([[s0]], [[s1]], [[s2]]) ← GetGenotypeDistr([[SDB]], k, [[ctrlmask]]);
    [[m1]] ← [[r0]] + [[r1]] + [[r2]];
    [[m2]] ← [[s0]] + [[s1]] + [[s2]];
    [[N]] ← [[m1]] + [[m2]];
    [[x]], [[y1]], [[y2]] ← 0;
    for i ∈ {0, 1, 2} do
        [[ni]] ← [[ri]] + [[si]];
        [[x]] ← [[x]] + αi ⊙ ([[ri]] ⊙ [[m2]] − [[si]] ⊙ [[m1]]);
        [[y1]] ← [[y1]] + (αi · αi) ⊙ [[ni]];
        [[y2]] ← [[y2]] + αi ⊙ [[ni]];
    [[x]] ← [[N]] ⊙ [[x]] ⊙ [[x]];
    [[y]] ← [[m1]] ⊙ [[m2]] ⊙ ([[N]] ⊙ [[y1]] − [[y2]] ⊙ [[y2]]);
    // Do correction due to fourfold discrepancy in counts;
    [[significance]] ← q ⊙ [[x]] ≥ 4p ⊙ [[y]];
return declassify([[significance]]);
```

The test statistic for the Cochran-Armitage test for trend can be expressed as

$$T_3 = \frac{N}{m_1 m_2} \cdot \frac{\left(\sum_{i=0}^{2} \alpha_i (r_i m_2 - s_i m_1)\right)^2}{N \cdot \sum_{i=0}^{2} \alpha_i^2 n_i - \left(\sum_{i=0}^{2} n_i \alpha_i\right)^2} \tag{7}$$

where the weights $\alpha_0, \alpha_1, \alpha_2$ are chosen according to the suspected influence mechanism. Algorithm 7 uses the data from Table 1 to calculate the test statistic T_3 based on equation (7). As additional inputs, Algorithm 7 is given weights α_0, α_1, and α_2. As mentioned in Subsection 3.2, results returned by Algorithm 3 are fourfold. Therefore, we can actually evaluate

$$x = \left(4N \cdot \sum_{i=0}^{2} \alpha_i (4r_i \cdot 4m_2 - 4s_i \cdot 4m_1)\right)^2 = 1024 \cdot N \cdot \left(\sum_{i=0}^{2} \alpha_i (r_i \cdot m_2 - s_i \cdot m_1)\right)^2$$

$$y = 4m_1 \cdot 4m_2 \cdot \left(4N \cdot \sum_{i=0}^{2} \alpha_i^2 4n_i - \left(\sum_{i=0}^{2} 4n_i \alpha_i\right)^2\right) = 256 \cdot m_1 m_2 \cdot \left(N \cdot \sum_{i=0}^{2} \alpha_i^2 n_i - \left(\sum_{i=0}^{2} n_i \alpha_i\right)^2\right)$$

and thus we must multiply y with 4 to correct the offset.

4.5 Transmission Disequilibrium Test

To calculate the test statistic for the transmission disequilibrium test (TDT), we need to know the count u of AA and the count v of BB genotypes among children. The statistic is computed as follows

$$T_4 = \frac{(u-v)^2}{u+v} \quad . \tag{8}$$

Algorithm 8: Transmission Disequilibrium Test

$\texttt{TDT}([\![\mathcal{SDB}]\!],\ k,\ \ell\ [\![\boldsymbol{childMask}]\!],\ p,\ q)$
begin
  $([\![\boldsymbol{u}]\!], [\![\boldsymbol{v}]\!]) \leftarrow \texttt{GetHomozygousChildCounts}([\![\mathcal{SDB}]\!], k, \ell, [\![\boldsymbol{childMask}]\!])$;
  $[\![\boldsymbol{x}]\!] \leftarrow [\![\boldsymbol{u}]\!] - [\![\boldsymbol{v}]\!]$;
  $[\![\boldsymbol{x}]\!] \leftarrow [\![\boldsymbol{x}]\!] \odot [\![\boldsymbol{x}]\!]$;
  $[\![\boldsymbol{y}]\!] \leftarrow [\![\boldsymbol{u}]\!] + [\![\boldsymbol{v}]\!]$;
  // Correction due to fourfold discrepancy in counts
  $[\![\boldsymbol{significance}]\!] \leftarrow q \odot [\![\boldsymbol{x}]\!] \geq 4p \odot [\![\boldsymbol{y}]\!]$;
  return $\texttt{declassify}([\![\boldsymbol{significance}]\!])$;

The counts u and v are obtained by using the Algorithm 4. Algorithm 8 uses this data to evaluate a test $T_4 \geq \frac{p}{q}$ using equation (8). As an additional input, Algorithm 4 is given the number of donors ℓ. Instead of two masks, the algorithm gets a zero-one mask $[\![\boldsymbol{childMask}]\!]$ for marking children in the case group. The mask vector is 3 times smaller than the donor count, as it is only applied to 1/3 of the donors' data as described in Algorithm 4.

4.6 Security analysis

Sharemind ensures that inputs and intermediate values remain secure throughout the computation. However, all the test algorithms presented in this Section have a single declassification call in the end of the algorithm. Each call declassifies a vector of boolean values where ones represent SNPs where the difference between the case and control groups was significant. Zeroes represent SNPs where no significant difference was observed. As the final declassified vector of zeroes and ones is equivalent to the desired list of significantly differentiated SNPs, the declassification reveals nothing beyond the desired outcome.

4.7 Multiple testing and FDR correction

In order to determine a proper lower bound $\frac{p}{q}$ for statistical tests, we must fix a significance level α. The standard significance threshold used in statistics is 0.05. As we do a genome-wise scan for finding significantly differentiated SNPs, we are in the multiple hypothesis testing scenario. As we know the total number of tested SNPs, we can apply Bonferroni correction, i.e, set $\alpha = \frac{0.05}{k}$ where k is the number of observed SNPs. The latter is often too harsh compared to FDR correction. Unfortunately, FDR is not so straightforward to apply in the privacy-preserving manner, as it works on p-values instead of easily computable test statistics.

Next, we will sketch how standard Benjamini-Hochberg procedure [BH95] can be implemented without leaking the individual test statistics. First, recall that the original BH-correction procedure first orders p-values in ascending order and then finds the largest i such that

$$p_{(i)} \leq \frac{i}{k}\alpha \tag{9}$$

where $p_{(i)}$ is the ith p-value and k is the total number of p-values. Second, note that all statistical tests we have devised for secure genome-wide association studies are one-tailed tests where the sampling distribution is independent from the size of the case and control group. As a result, we can reformulate the problem in terms of test statistics.[1]

[1]Analogous methodology works also for two-tailed tests or in the case where the sampling distribution depends

Algorithm 9: Privacy-preserving Benjamini-Hochberg procedure

`SecureBHTesting`($[\![\boldsymbol{u}]\!]$, $[\![\boldsymbol{v}]\!]$, α)

begin

Form triples $([\![u_1]\!], [\![v_1]\!], [\![1]\!]), ([\![u_2]\!], [\![v_2]\!], [\![2]\!]), \ldots, ([\![u_k]\!], [\![v_k]\!], [\![k]\!])$.

Use oblivious sorting to sort triples in descending order wrt to secret ratios $\frac{u_i}{v_i} = t_i$.

Let $([\![u_{(1)}]\!], [\![v_{(1)}]\!], [\![c_{(1)}]\!]), ([\![u_{(2)}]\!], [\![v_{(2)}]\!], [\![c_{(2)}]\!]) \ldots, ([\![u_{(k)}]\!], [\![v_{(k)}]\!], [\![c_{(k)}]\!])$ be the result.

Compute fractional approximations $\frac{p_i}{q_i} \approx Q_{\text{upper}}\left(\frac{i\alpha}{k}\right)$ from public information.

Evaluate significance tests $t_i \geq Q_{\text{upper}}\left(\frac{i\alpha}{k}\right)$ in parallel: $[\![\boldsymbol{s}]\!] \leftarrow \boldsymbol{p} \odot [\![\boldsymbol{v}_*]\!] \leq \boldsymbol{q} \odot [\![\boldsymbol{u}_*]\!]$.

Start opening values $s_k, s_{k-1}, \ldots s_1$ until the first one $s_{i_*} = 1$ is revealed.

Open the locations $c_{(1)}, \ldots, c_{(i_*)}$ and declare corresponding SNPs significant.

More precisely, note that all statistical tests for GWAS use the upper tail of χ^2-distribution with one degree of freedom to compute the p-value. Hence, the correspondence between p-values and test statistics is anti-monotone, we can consider decreasingly ordered test statistics $t_{(1)}, \ldots, t_{(k)}$ instead of p-values:

$$p_{(i)} \leq \frac{i}{k}\alpha \qquad \Longleftrightarrow \qquad t_{(i)} \geq Q_{\text{upper}}\left(\frac{i\alpha}{k}\right) \enspace ,$$

where Q_{upper} denotes the upper quantile of the χ^2-distribution

$$Q_{\text{upper}}(\alpha) = t_0 \qquad \Longleftrightarrow \qquad \Pr\left[t \geq t_0\right] = \alpha \enspace .$$

As the significance threshold α and the function Q_{upper} are public, we publicly compute fractional approximations of significance thresholds

$$\frac{p_{(i)}}{q_{(i)}} \approx Q_{\text{upper}}\left(\frac{i\alpha}{k}\right)$$

and use secure computing to evaluate comparisons $t_{(i)} \geq \frac{p_{(i)}}{q_{(i)}}$.

Let the test statistic t_i be represented as a fraction $\frac{u_i}{v_i}$ and let $\boldsymbol{u}$ and $\boldsymbol{v}$ be the corresponding vectors for denominator and numerator values. Then Algorithm 9 depicts the corresponding privacy-preserving significance testing procedure, which reveals only the locations c_i and ordering of significant SNPs. It is even possible to hide the ordering if the shares $[\![c_{(1)}]\!], \ldots, [\![c_{(i_*)}]\!]$ are obliviously shuffled before opening such that $c_{(1)}, \ldots, c_{(i_*)}$ are opened in unknown random order.

The most complex step in this algorithm is the oblivious sorting step which rearranges triples in descending order according to the ratios $t_i = \frac{u_i}{v_i}$. This is usually implemented by using oblivious compare-exchange blocks which securely implement the following functionality:

$$\textsc{OblCompEx}((u_1, v_1, c_1), (u_2, v_2, c_2)) = \begin{cases} (u_1, v_1, c_1), (u_2, v_2, c_2), & \text{if} \quad u_2 v_1 \leq u_1 v_2 \enspace , \\ (u_2, v_2, c_2), (u_1, v_1, c_1), & \text{if} \quad u_2 v_1 > u_1 v_2 \enspace . \end{cases}$$

Secure evaluation of such block requires 8 multiplications and 1 comparison. One of the best parallel sorting networks is the pairwise sorting network that needs $n + \frac{n(\log_2 n)(\log_2 n - 1)}{4} - 1$ such OblCompEx gates [Par92]. In our case, this mean that secure oblivious sorting requires approximately 162.6 million secure multiplication and 20.3 million secure comparison operations. A back-of-the envelope estimate based on Sharemind performance data shows that this would roughly take 10 minutes [BNTW12].

on the sizes of case and control groups. However, a general solution for these cases contains tedious technical details that are not relevant in our context of genome-wide association studies.

Algorithm 10: Privacy-preserving block-deletion detection

```
SecureDeletionDetection([[SDB]], k, t, δ)
begin
    for i ∈ {0, ..., ⌊k/t⌋} do
        [[bsum]][i, ∗] ← Σ_{a=2δ+it}^{2δ+(i+1)t} [[SDB]][a, ∗];
        [[deletions]] ← [[bsum]] = 0;
    return [[deletions]];
```

4.8 Optimisations for the FDR correction procedure

In most cases, we do not expect to see a large number of significant SNPs. In fact, lists that are over 1000 elements are hard to interpret and follow up. Hence, we could consider more conservative BH-correction procedure that always outputs a list of at most 1000 elements with false discovery rate below α. That is, we want to find the largest $i \leq 1000$ that still satisfies the equation (9). The latter is a much simpler task, as we must first find the top 1000 elements which can be done with $O(k)$ comparisons. After that we must still sort the top 1000 elements but this takes only approximately 26000 secure comparisons and thus significantly speeds up the FDR-correction so that the running time can me measured in seconds instead of minutes.

Another alternative is to reveal all test statistics in random order so that we can perform the BH-correction with public values but at the same time we will not know which test statistic corresponds to which SNP location. This can be achieved with oblivious shuffle for which efficient protocols are known [LWZ11]. Performance benchmarks on shuffling show that for our data size, shuffling can be completed in under a minute. After the appropriate threshold is known, the list of relevant SNPs can be detected as usual.

To conclude, FDR correction is feasible if we control the maximal output size or make trade-offs between privacy and efficiency. However, the exact details and performance results are out of the scope of this manuscript.

5 Privacy-Preserving Methods for Quality Control

In this section, we describe example methods for detecting anomalies in the genomic data, which might influence the results of genome-wise association studies. These simplified examples are just meant to show what is possible, how much time it takes and what trade-offs are possible between performance, power for anomaly detection and privacy.

5.1 Algorithms for Monitoring Data Quality

A human genome can have long regions that are missing (the so-called genomic deletions) compared to the reference genome. Overrepresentation of such deletions in the selected study group can skew the test results or be an interesting fact itself. Due to the measurement procedure, such deletions induce NN measurements for SNPs located in the region of deletion, as probes for these SNPs have no complementary DNA material to hybridize. In our setting, NN measurements are represented as $(0, 0)$ pairs. Consequently, an entire block of SNPs is missing if and only if the corresponding block of indicator variables sums to zero. Algorithm 10 extends this idea and performs this test in parallel for many blocks at the same time.

The algorithm takes as input the block length t and offset δ and outputs secret-shared indicator values *deletions* $[i, j]$ that show whether the ith block with offset δ of the jth donor is a streak of NN pairs. As the result is shared this result can be further processed. For instance, we can compute the number of deletions for the selected set of individuals by evaluating

$$\sum(\llbracket \boldsymbol{mask} \rrbracket \odot \llbracket \boldsymbol{deletions} \rrbracket [i, *]), \qquad i \in \{0, \ldots, \lfloor k/t \rfloor\}$$

or testing if this sum exceeds a certain threshold.

These types of tests are not computationally intensive, as additions are extremely cheap and the number of comparisons is proportional to the number of entries in $\mathcal{SDB}$. As Sharemind can perform tens of thousands of secure tests in a second, we estimate that such a deletion detection will take under a minute [BNTW12].

The second common problem in SNP measurements is (geographic) variability in allele frequencies. Although each SNP commonly has only two alleles: a basic form and its point mutation, in some subpopulations mutations are different. Hence, one often filters out SNPs for which the SNP is constant – one allele is almost omnipresent in all individuals or frequency of both alleles is below a certain threshold. These conditions are straightforward to test. For that, we first compute frequencies of both alleles or allele pairs using Algorithms 2 and 3. Given the frequencies, we can use thresholding to detect whether counts of both alleles are over certain threshold or not. Again, since comparisons are not too expensive, this operation would not take more than a few minutes.

5.2 Examples of Outlier Detection and Filtering Algorithms

Note that there are two ways how to use results of various data quality checks. First, we can publish the end results and study them as in usual data analysis projects. The second option is to use corresponding zero-one index vectors to filter out invalid outputs without leaking which SNPs or SNP-blocks are invalid. This option is required when the results of the data quality tests leak private information. For instance, the fact that a certain block deletion occurs in the study might reveal the identity of a donor, when this is a rare deletion.

In the following, we sketch how oblivious filtering can be used in the context of phenotypic data quality indicators. It can be analogously applied also for the genotypic indicators discussed above by switching rows and columns. Let vector $\boldsymbol{x}$ consist of measurements of a specific phenotypic trait such a blood pressure. Then we might want to exclude patients with extreme values of blood pressure from the study. For blood pressure, we can define the threshold based on background knowledge, e.g., declare patients with systolic blood pressure over 170 mmHg inapplicable for the study. In general, we might try to compute the threshold based on standard deviation or on 5% and 95% quantiles. As the variance can be expressed in terms of simple arithmetic operations

$$\textsc{Var}(\boldsymbol{x}) = \frac{1}{n}\sum_{i=1}^{n}(x_i - \overline{x})^2 = \frac{1}{n^3}\sum_{i=1}^{n}\left(nx_i - \sum_{i=1}^{n} x_i\right)^2 \quad ,$$

it is straightforward to compute shares of $n^3\textsc{Var}(\boldsymbol{x})$. Similarly, we can convert the outlier criterion $|x_i - \overline{x}| \leq \alpha\sqrt{\textsc{Var}(\boldsymbol{x})}$ into an equivalent condition without division operations

$$n\left(nx_i - \sum_{i=1}^{n} x_i\right)^2 \leq \alpha^2 \sum_{i=1}^{n}\left(nx_i - \sum_{i=1}^{n} x_i\right)^2 \quad .$$

Note that this condition can be securely evaluated even if the size n is in secret shared form. Computational complexity of these computations is marginal, as each test requires three secure multiplication and a single comparison operation.

The simplest way to find out upper and lower quantiles is to use oblivious sort. For instance, if the vector $\boldsymbol{x}$ has 1000 elements then elements $x_{(50)}$ and $x_{(950)}$ correspond to lower and upper 5% quantiles. Of course, we can use more efficient algorithms for computing the quantiles. However, even with oblivious sort this step is practically feasible for 10000 individuals. In more elaborate cases, the the quantile falls between two elements $x_{(i)}$ and $x_{(i+1)}$ and we have to use standard interpretation formulae. But this does not significantly increase the complexity. Assume that we have obtained $[\![q_0]\!]$ and $[\![q_1]\!]$. Then outlier detection requires two comparisons and one multiplication per vector element:

$$[\![\boldsymbol{omask}]\!] \leftarrow ([\![\boldsymbol{x}]\!] \leq [\![q_0]\!]) \odot ([\![\boldsymbol{x}]\!] \geq [\![q_1]\!]) \ .$$

The simplest way to use the outlier mask is to combine it with the group selection mask in order to exclude persons with outlier measurements from the study. Let $[\![\boldsymbol{mask}]\!]$ be the mask that was originally used to determine the case or control group. Then we can just update it

$$[\![\boldsymbol{mask}]\!] \leftarrow [\![\boldsymbol{mask}]\!] \odot (1 - [\![\boldsymbol{omask}]\!]) \ .$$

If the $[\![\boldsymbol{omask}]\!]$ lists SNPs with dubious quality then there are two ways how to use it. First, we can just update the indicator vector for the significant SNPs

$$[\![\boldsymbol{significance}]\!] \leftarrow [\![\boldsymbol{significance}]\!] \odot (1 - [\![\boldsymbol{omask}]\!])$$

before declassification. Alternatively, we can eliminate corresponding rows of the $\mathcal{SDB}$ by using oblivious filtering techniques [LWZ11]. This approach is useful in conjunction with FDR-correction, as it reduces the number of performed statistical tests and thus increases the statistical power. As the oblivious filtering is roughly equivalent to oblivious database shuffle, then it is clearly feasible even for 300000 SNPs [LWZ11].

6 Performance evaluation

We tested all algorithms by splitting the set of donors into two equal parts. In biology, this is a rather uncommon way to test genome-wide association algorithms, as the relevance of the results greatly depends on how well the case and control groups are chosen. For our purposes, however, this strategy is as good as any other, as we do not test the statistical properties of the algorithms. One can wonder whether algorithms will run slower if we choose the case and control groups differently. The latter is impossible, as the number of operations depends only on the size of the SNP database. In fact, the algorithm would be insecure if its running-time depended on a group's size or its members.

References

[BH95] Y. Benjamini and Y. Hochberg. Controlling the False Discovery Rate: A Practical and Powerful Approach to Multiple Testing. *Journal of the Royal Statistical Society. Series B (Methodological)*, 57(1):289–300, 1995.

[BLW08] Dan Bogdanov, Sven Laur, and Jan Willemson. Sharemind: A Framework for Fast Privacy-Preserving Computations. In *Proceedings of ESORICS'08*, volume 5283 of *Lect Notes Comput Sc*, pages 192–206, Heidelberg, 2008. Springer.

[BNTW12] Dan Bogdanov, Margus Niitsoo, Tomas Toft, and Jan Willemson. High-performance secure multi-party computation for data mining applications. *International Journal of Information Security*, 11(6):403–418, 2012.

[Jag10] Roman Jagomägis. SecreC: a Privacy-Aware Programming Language with Applications in Data Mining. Master's thesis, Univesity of Tartu, 06 2010.

[LWZ11] Sven Laur, Jan Willemson, and Bingsheng Zhang. Round-efficient oblivious database manipulation. In Xuejia Lai, Jianying Zhou, and Hui Li, editors, *ISC*, volume 7001 of *Lecture Notes in Computer Science*, pages 262–277. Springer, 2011.

[Par92] Ian Parberry. The Pairwise Sorting Network. *Parallel Processing Letters*, 02(02n03):205–211, 1992.
